# Assessment of ultraviolet and infrared radiation transmission through automobile windshields and side windows

**DOI:** 10.3389/fpubh.2024.1497357

**Published:** 2025-04-15

**Authors:** Nouf Jubran AlQahtani, Ghada Naje AlEssa, Hoor Fayez AlDushaishi, Amnah Nabil Bukair, Syed Mehmood Ali

**Affiliations:** Biomedical Engineering Department, College of Engineering, Imam Abdulrahman Bin Faisal University, Dammam, Saudi Arabia

**Keywords:** solar radiation, ultraviolet (UV), infrared (IR), skin damage, skin cancer, vehicle, data logger radiometer

## Abstract

**Introduction:**

Excessive exposure to solar radiation, particularly ultraviolet (UV) and infrared (IR) rays, poses significant health risks, including skin damage and an increased risk of skin cancer. While the penetration of UV radiation through vehicle windows is well-documented, the potential transmission of IR radiation remains less recognized.

**Methods:**

A total of 1,293 participants participated in a survey to assess awareness of solar radiation risks and protective behaviors, revealing a notable lack of attention to protective measures despite widespread knowledge of the risks associated with solar exposure. This study investigates UV and IR radiation exposure inside and outside vehicles in the Middle East, a region known for its extreme temperatures exceeding 52°C. Radiation levels were measured using a PMA2100 data logger radiometer in 20 vehicles.

**Results and discussion:**

The results demonstrated the ability of both UV and IR radiation to transmit through vehicle windows. For instance, the highest UV transmission through the side and front windows were recorded at 1.70 and 0.80 mW/cm^2^, respectively, while the IR transmission through the side and front windows were 84.17 and 98.27 mW/cm^2^, respectively. These findings highlight the need for improved protective measures against both UV and IR radiation, especially in hot climates where prolonged exposure to solar radiation is common. The study also identifies a gap in public awareness of IR radiation and calls for further research into effective strategies for mitigating these risks.

## 1 Introduction

Sunlight is essential for life; however, excessive exposure can lead to significant health consequences. Prolonged exposure to solar radiation is associated with severe skin damage, skin cancer, and ocular disorders. Solar radiation consists of three primary components: ultraviolet (UV; 280–400 nm), infrared (IR; 760 nm−1 mm), and visible light (VL; 400–760 nm) (1). Both UV and IR radiation can disrupt physiological functions, resulting in various health issues. UV radiation is the leading cause of sunburn, skin cancer, and premature skin aging (2). In contrast, IR radiation is primarily associated with skin photoaging and can contribute to conditions such as erythema ab igne (3). Emerging epidemiological evidence suggests that IR exposure may also play a role in the development of skin cancer (4). Furthermore, IR radiation increases skin temperature to ~43°C, which is then converted into heat energy, leading to significant thermal damage to skin tissues (5).

An inverse relationship exists between energy and wavelength. Shorter wavelengths, such as UV radiation, carry higher energy than longer wavelengths, such as IR radiation. The higher energy of UV radiation enables it to cause more immediate damage to the skin's surface layers, although this also limits its depth of penetration. UV radiation primarily affects the epidermis, where its high energy is absorbed by the outer layers of the skin, leading to surface-level damage such as sunburn and skin cancer (6). In contrast, IR radiation, with its longer wavelength and lower energy, penetrates more deeply into the skin. Longer wavelengths are less efficiently absorbed by the outer layers, allowing them to travel further into the dermis. While IR radiation has lower energy than UV radiation, its greater depth of penetration can result in significant tissue damage over time (1).

Numerous studies have highlighted the adverse effects of prolonged exposure to solar radiation on human health (7–11). Therefore, monitoring UV and IR radiation levels is crucial, as excessive exposure can result in harmful effects. To assess the risks associated with UV and IR radiation, two commonly used metrics are the UV Index (UVI) and IR irradiance. The UVI quantifies the intensity of UV radiation, with each level corresponding to a different degree of risk and need for protection. The UVI is a dimensionless scale that ranges from 0 (indicating no UV radiation) to +11 (extremely dangerous levels). A UVI of 3 or higher can result in health issues, such as skin damage and cancer (12). In contrast, irradiance measures the amount of solar energy incident on a surface, with higher irradiance values indicating greater radiation levels. Cumulative exposure to UV and IR radiation poses a greater risk than a single exposure.

Although the harmful effects of UV and IR radiation are well-known, it is not feasible to completely avoid exposure to these radiation types, despite the associated risks. Therefore, significant attention has been directed toward the development of protective technologies and strategies to mitigate the harmful impact of solar radiation. Common protective measures include the use of sun protection factor (SPF) products and the wearing of ocular filters such as sunglasses. However, these approaches provide only partial protection. As a result, there is a growing interest in the development of personalized monitoring devices to more effectively assess and manage exposure. For instance, Amini et al. (13) engineered a UV monitoring device integrated with specialized software capable of recording an individual's UV exposure history and cumulative dosage over periods ranging from a day to a month. Shi et al. (14) introduced an ultra-thin, stretchable wearable UV sensor designed to quantify both UV exposure and the effectiveness of sunscreen. This sensor is sufficiently flexible to be worn continuously for up to 5 days, even during normal daily activities. Furthermore, AlQahtani et al. (15) developed a novel wearable device intended to provide protection from both UV and IR radiation, offering efficacy in both indoor and vehicular environments.

**Figure 1:**
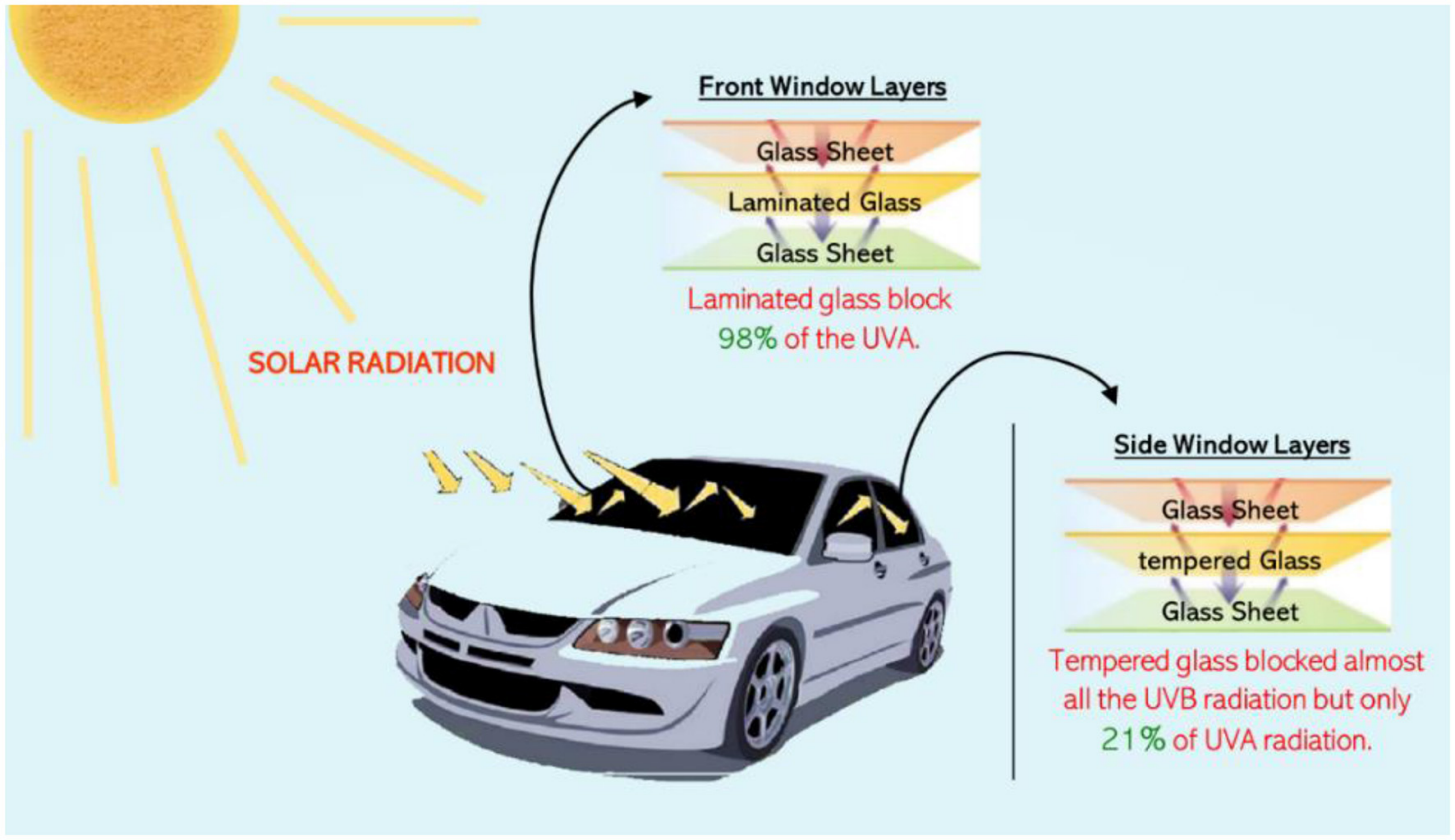
Solar radiation and vehicle window design: a comparison of UV and IR transmission through different types of vehicle glass.

Although some individuals can avoid extreme UV and IR radiation exposure during peak hours, outdoor workers, such as drivers, face constant exposure. Therefore, it is crucial that vehicle windows provide effective protection against these radiation types. Vehicles typically employ two main types of glass: laminated and tempered, as illustrated in Figure 1. Laminated glass consists of multiple layers of glass with an interlayer of polyvinyl butyral (PVB), a synthetic, non-degradable polymer commonly used in windshields to reduce UV radiation transmission (16). In contrast, tempered glass, designed to enhance safety by breaking into small, less hazardous pieces during accidents, is typically used for side and rear windows. However, tempered glass transmits higher levels of UV radiation because it lacks a PVB interlayer (17).

Several studies have demonstrated that UV radiation can penetrate vehicle windows, exposing drivers to harmful radiation during driving (6, 18–21). However, the potential risks posed by IR radiation inside vehicles have received limited attention. Despite its often-underestimated significance, IR radiation is not effectively mitigated in most vehicles, as evidenced by the lack of glass specifically designed to block it. For instance, Boxer Wachler (20) reported that side windows transmit 25% more UV radiation compared to front windows. Moreover, Duarte et al. (22) showed that UV radiation can still pass through laminated glass, even in the presence of the PVB interlayer. Both PVB interlayers and IR coatings influence the amount of radiation transmitted through windows, with varying degrees of effectiveness. IR coatings, typically composed of metal-based materials, allow controlled transmission of IR radiation while reflecting higher proportions. Despite their potential efficacy, these coatings are not widely adopted in vehicle manufacturing due to their increased cost.

Other studies have indicated that the areas of the driver's body nearest to the window are more vulnerable to UV and IR radiation damage due to continuous exposure (24–26). For instance, a study conducted by researchers at Washington University found a higher rate of skin cancer on the left side of the body, which they attributed to the study being conducted in the United States, a left-driving country. They also noted an increased incidence of skin cancer on the upper left arm, a region commonly exposed to solar radiation while driving (24). Another study in Australia, a right-driving country, found a higher incidence of skin cancer on the right side of the body (25).

This study aims to assess solar radiation exposure within vehicles, specifically quantifying the levels of UV and IR transmission through vehicle windows. Given the significant amount of time drivers spend inside vehicles, it is essential that these vehicles effectively shield occupants from harmful UV and IR radiation. The primary objective is to evaluate the extent of UV and IR exposure while driving. The findings from this study will contribute to the development of strategies aimed at reducing solar radiation exposure both inside and outside vehicles, along with mitigating its associated health risks. To the best of our knowledge, this study is the first of its kind to comprehensively evaluate both UV and IR radiation exposure within vehicles in the Kingdom of Saudi Arabia (KSA).

## 2 Methods

### 2.1 Experimental framework

A sequential experiment was conducted on twenty vehicles using the Solar Light Model PMA2100 Dual-Input Data Logging Radiometer, a device capable of measuring both UV and IR radiation transmitted through vehicle windows. Prior to the experiment, a local awareness assessment regarding the risks associated with solar radiation exposure was carried out. A survey was administered to evaluate public knowledge of the potential health effects of solar radiation in the region. The survey covered aspects such as participants' demographics, country of residence, understanding of solar radiation risks, and their practices regarding sun avoidance and preventive measures.

**Figure 2:**
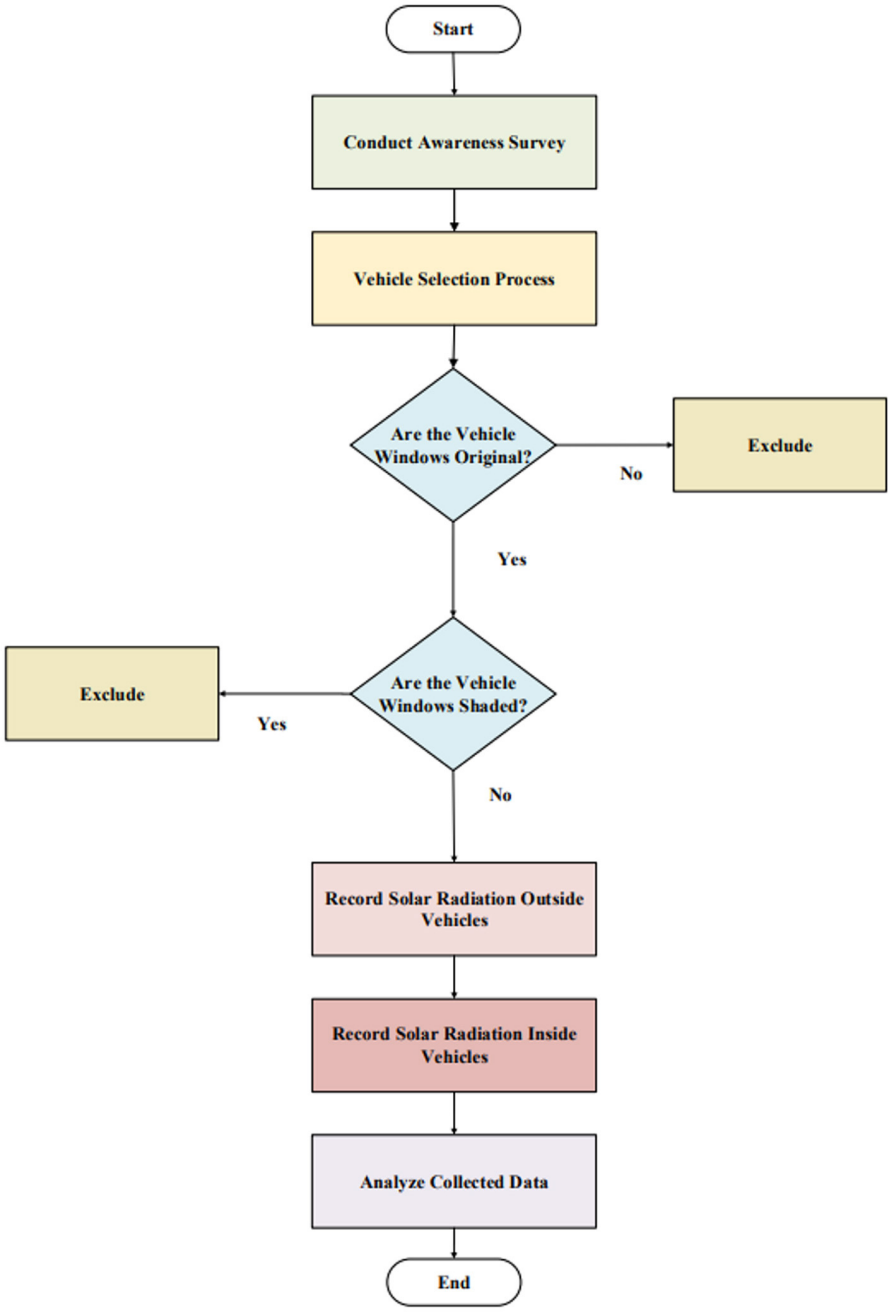
Outlining the steps followed in this study through an experimental framework.

The experiment to assess solar protection levels was conducted in Dammam, Saudi Arabia, on sunny days in June 2021, between 9:00 a.m. and 3:00 p.m. Dammam, characterized by a desert climate, has an average annual temperature of 26.4°C. The inclusion criteria for vehicles were as follows: (i) the vehicle windows must be original (i.e., not replaced due to accidents) and (ii) the vehicles must not be shaded. The experimental framework followed in this study is illustrated in Figure 2.

### 2.2 Sensors and measurements

**Figure 3:**
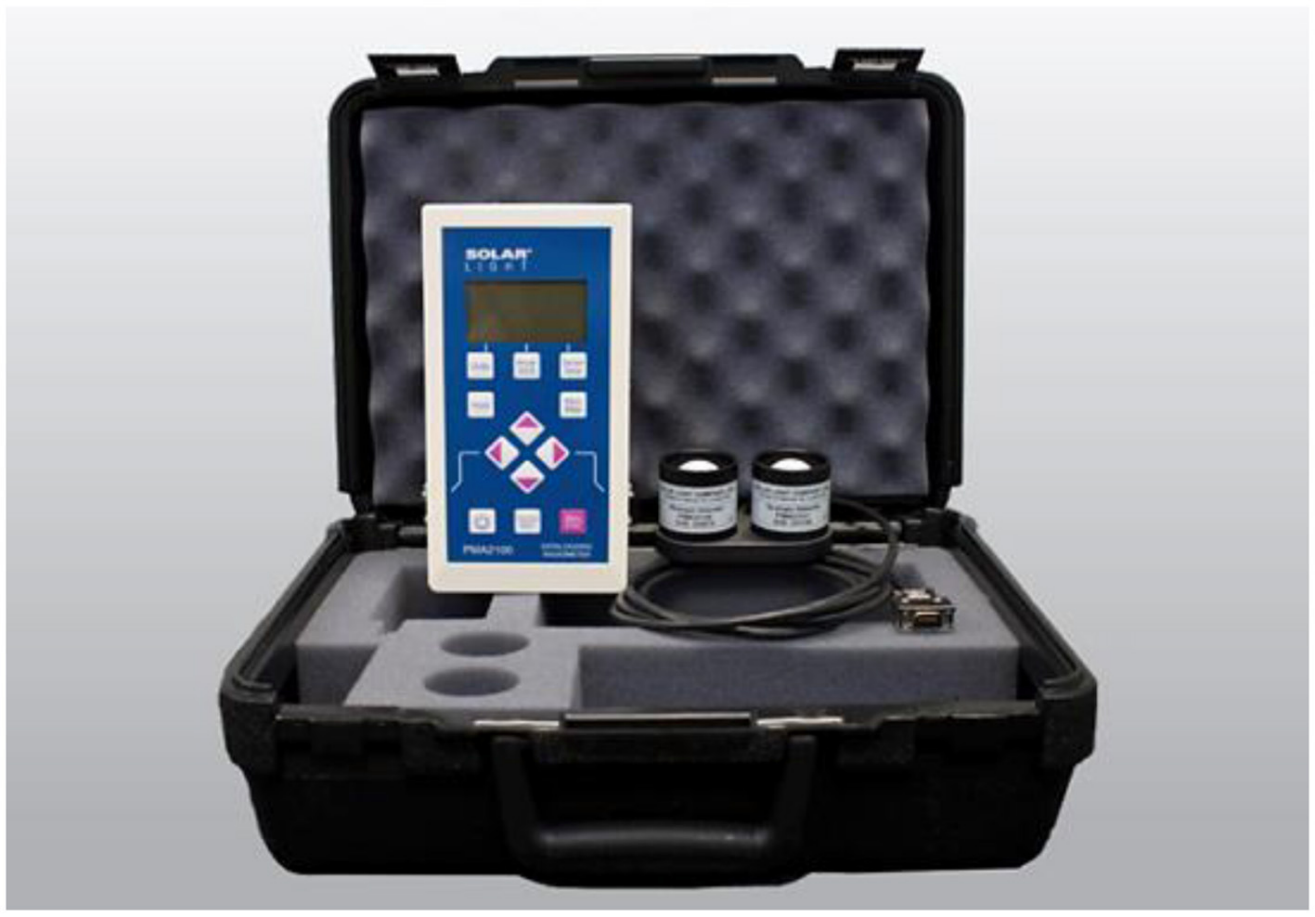
Data logger radiometer and sensors.

The Solar Light Model PMA2100 Dual-Input Data Logging Radiometer (shown in Figure 3) was used in this study to precisely measure solar radiation. This instrument incorporates two distinct sensors: the PMA2140 and PMA2107, which enable the simultaneous measurement of different spectral components of solar radiation, specifically UV and IR irradiance.

The PMA2107 sensor is designed to measure UV irradiance, which is important for assessing the potential risks of UV-induced damage to human health due to prolonged exposure. Although UV radiation constitutes a relatively small fraction of the solar spectrum, it is highly energetic and can have significant biological effects, including skin damage and an increased risk of skin cancer. In contrast, the PMA2140 sensor is optimized for detecting IR irradiance, a key component of solar radiation that primarily contributes to thermal energy. This sensor measures the intensity of IR radiation, which plays a major role in heating skin and inducing thermal damage.

In this study, the PMA2100 data logger was employed to record irradiance values in units of milliwatts per square centimeter (mW/cm^2^), enabling a detailed and quantitative analysis of radiation levels both inside and outside of vehicles. The dual-input functionality of the PMA2100 allowed for continuous monitoring of both UV and IR irradiance, while its data logging capabilities stored measurements for subsequent analysis. This made the PMA2100 an invaluable tool for investigating solar radiation exposure within vehicles. It was particularly useful for assessing the effectiveness of vehicle window tints in blocking harmful UV and IR radiation and for evaluating the potential impacts of solar radiation on human skin.

### 2.3 Vehicle windows and sensor placement

Vehicle windows are fabricated from laminated and tempered glass, as previously mentioned. While the general material composition of the windows is known, the specific manufacturing processes and design parameters for each vehicle are not publicly disclosed. Automotive manufacturers typically consider such information proprietary, including details on manufacturing techniques and window specifications. Therefore, although the windows in this study are composed of the same general materials (laminated and tempered glass), there may be inherent variations in the design and construction of individual windows across different vehicle models.

In addition to material composition, there may be differences in other critical factors, such as glass thickness, the incorporation of UV-blocking coatings, the degree of tinting, and other special treatments. These factors could influence the transmission of solar radiation, specifically UV and IR radiation, through the windows. However, due to the lack of detailed, vehicle-specific data on window construction, the precise characteristics of the individual windows in this study could not be fully determined. These potential variations may impact the level of UV and IR radiation transmitted through the windows.

The sensors of data logger radiometer were positioned to measure solar irradiance levels both inside and outside of the vehicles. Solar irradiance measurements were initially recorded for 16 min outside the vehicles under direct exposure to solar radiation. After the external measurements, irradiance was then recorded for 16 min inside each vehicle, with the sensors fixed in place to ensure consistency throughout the experiment. The measurements were taken stationary vehicle, and the measurements were recorded in each vehicle between 9:00 a.m. and 3:00 p.m., indicating that the measurements were taken within this time period, but we did not measure the solar irradiance in 20 vehicles simultaneously.

Since the experiment was conducted in the KSA, a left-driving country, the PMA2107 and PMA2140 sensors were strategically placed on the left side window and the front window of each vehicle to capture the solar radiation exposure accurately. We precisely placed these sensors in direction of solar radiation.

The PMA2107 sensor, which measures UV irradiance, was positioned to capture direct sunlight entering through the vehicle windows, while the PMA2140 sensor, designed to measure IR irradiance, was placed similarly to assess the infrared component of solar radiation. This setup enabled simultaneous monitoring of both UV and IR irradiance levels in the vehicle interior.

### 2.4 Statistical analysis

The collected data were subjected to rigorous statistical analysis to draw meaningful conclusions regarding solar radiation exposure both inside and outside the vehicles. The analysis aimed to characterize the distribution and intensity of UV and IR radiation levels. Survey results were evaluated and presented using a range of visualization tools, including bar graphs and scatter plots. These graphical representations facilitated the assessment of awareness levels related to radiation exposure. In addition to graphical analysis, descriptive statistics were employed to analyze the UV and IR radiation data. Key summary statistics, including the minimum, maximum, mean, and standard deviation (STD), were calculated. The minimum and maximum values provided the range of observed radiation levels, while the mean offered an estimate of the central tendency for UV and IR irradiance both inside and outside the vehicles. The standard deviation was computed to assess the variability in radiation measurements, offering insight into the consistency of exposure levels across different time intervals and vehicle conditions.

## 3 Results

### 3.1 Survey data

The survey aimed to assess participants' awareness of solar radiation exposure, collecting responses from a total of 1,293 individuals. Participants were asked to provide information on their country of residence, their information of the risks associated with solar radiation, and their practices regarding sun avoidance and protective measures.

The results revealed that the majority of participants (~66.82%) were residents of Kingdom of Saudi Arabia. In terms of age distribution, 51% of respondents were over the age of 41, while 23% were between the ages of 21 and 30, 14% were between 31 and 40 years old, and 12% were between 15 and 20 years old.

**Figure 4:**
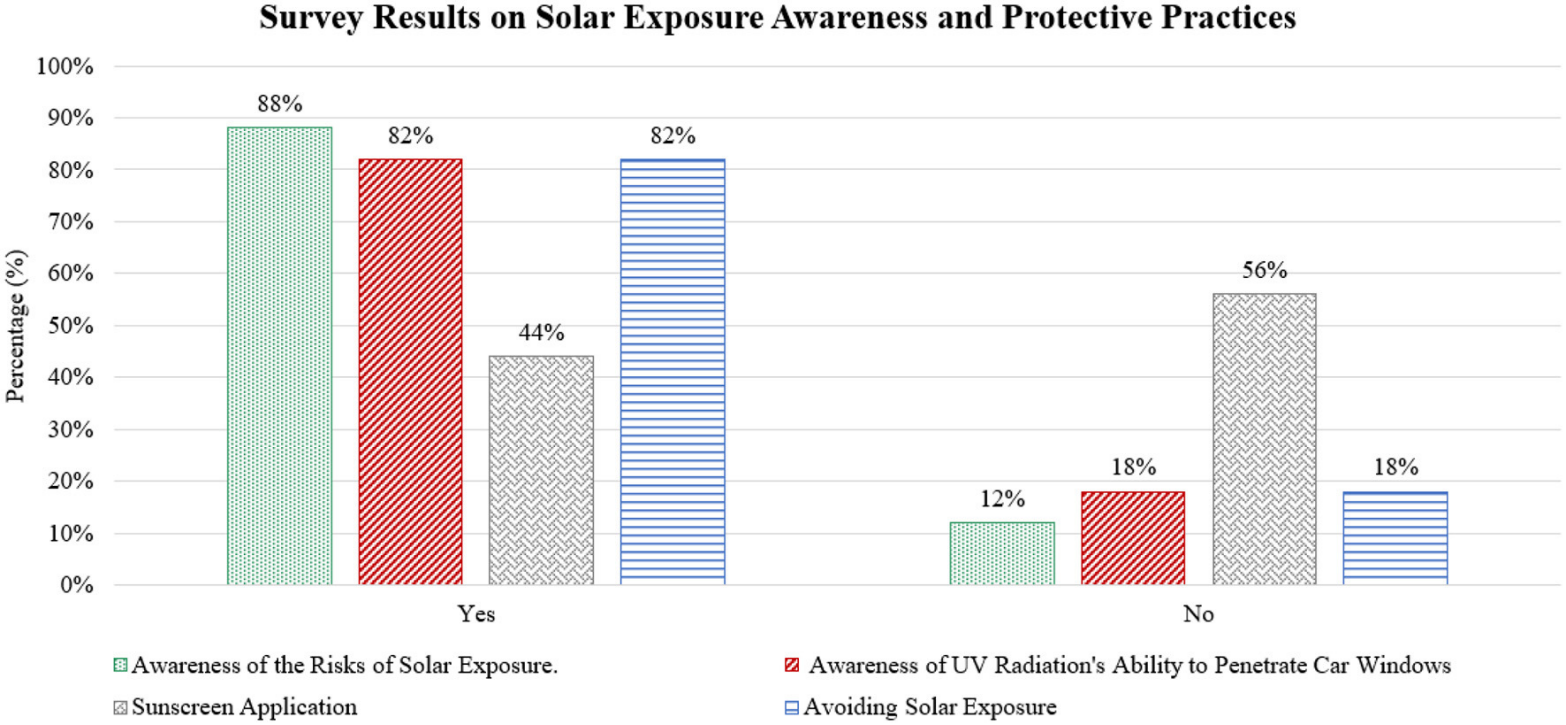
Survey results on awareness of UV exposure risks and protective measures.

Further analysis of the data on participants' awareness levels and their practices regarding solar radiation protection measures is presented in Figure 4.

### 3.2 UV irradiance levels

**Figure 5:**
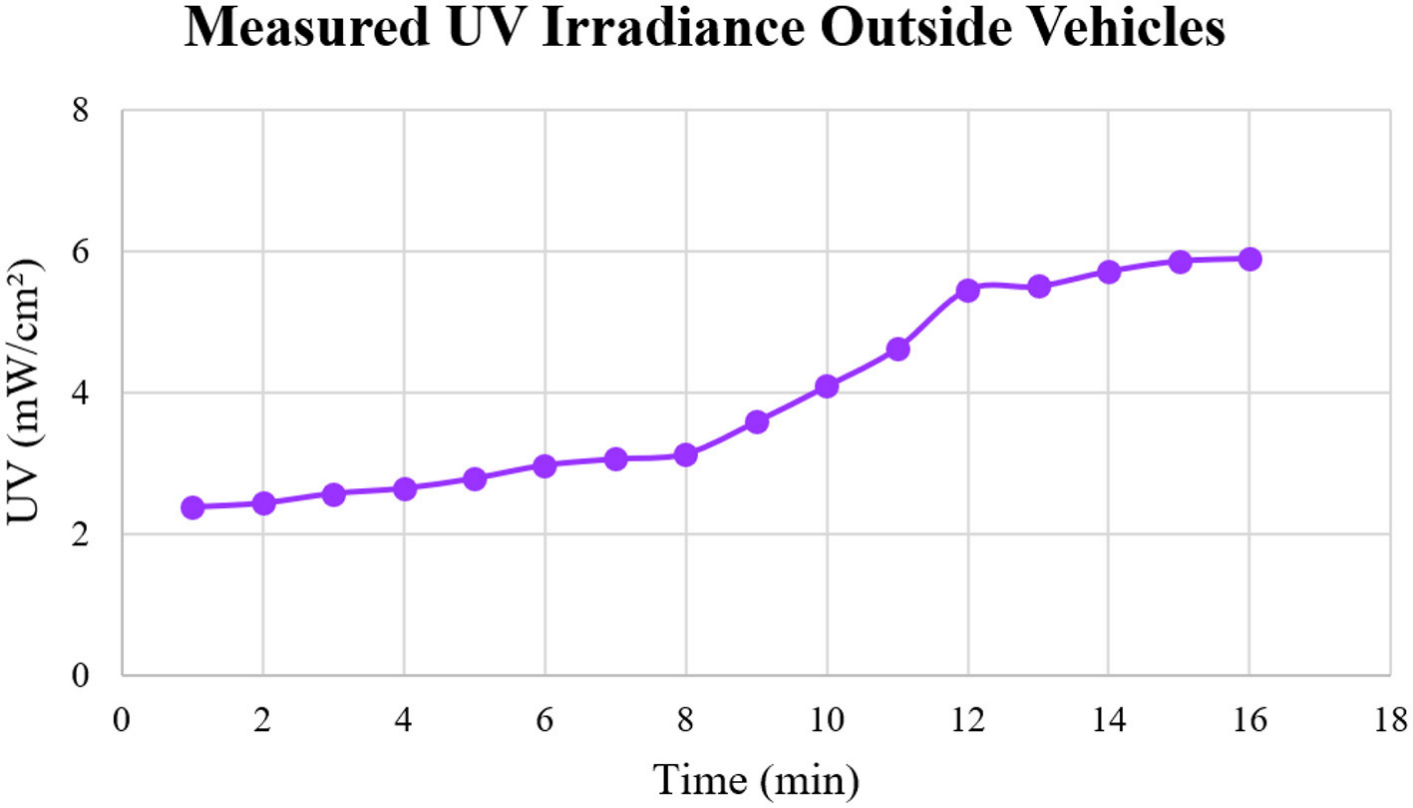
UV measurements outside the vehicles for 16 min.

**Figure 6:**
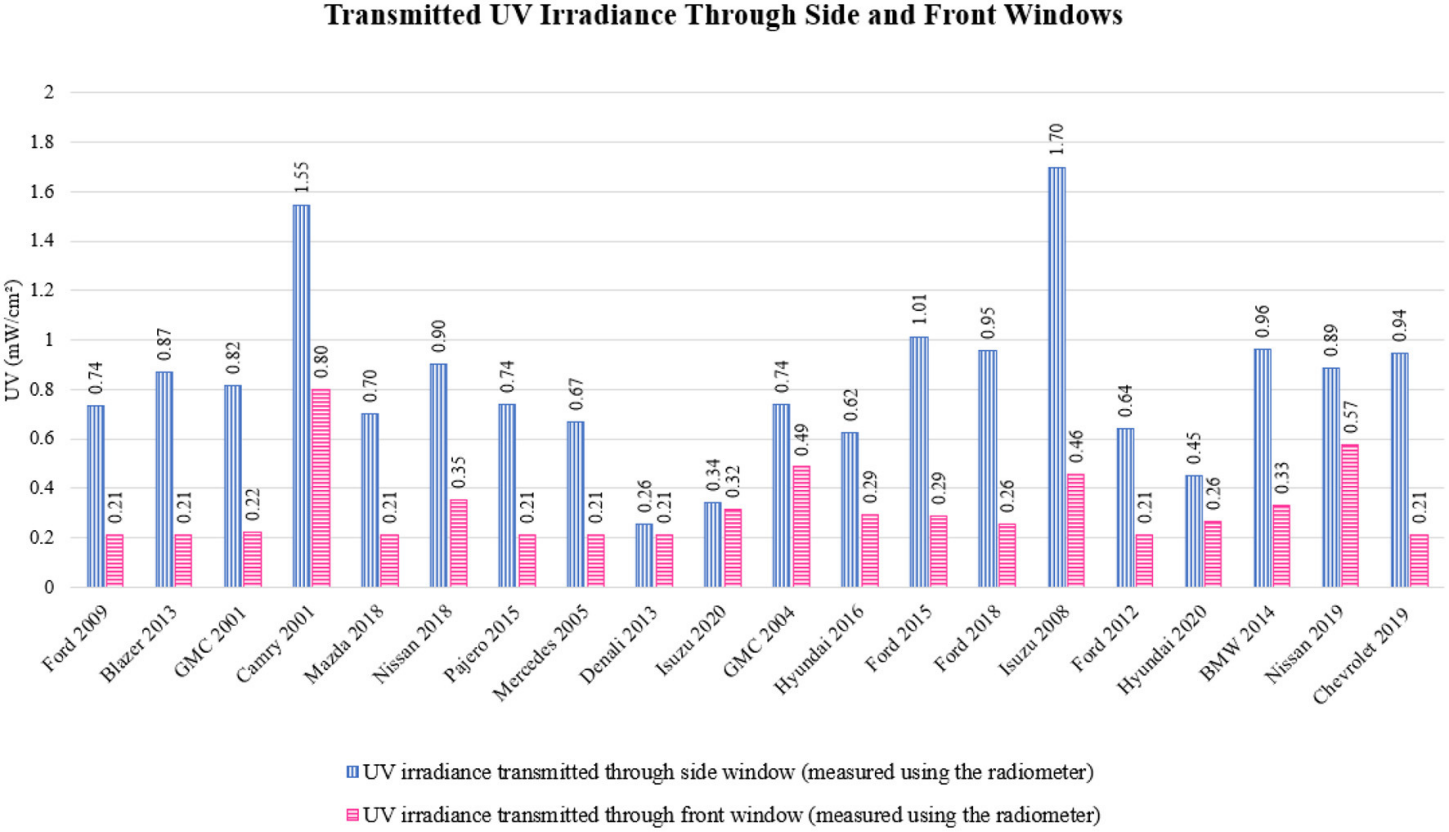
Transmitted UV irradiance through the side (represented in green color) and front (represented in blue color) windows in 20 vehicle models; the measurements were taken using the radiometer.

UV irradiance measurements were collected to evaluate the level of solar radiation transmitted through the vehicle windows, providing insights into the potential exposure to UV radiation within the vehicle. The recorded UV irradiance levels were analyzed across all vehicles, with particular emphasis on variations in transmission between different window types (i.e., side windows and front windows). The analysis revealed significant differences in UV irradiance between vehicles, with variations attributed to factors such as vehicle model, window material, and the presence of tinting or other protective coatings. Figure 5 illustrates the UV irradiance levels measured outside the vehicles, while Figure 6 displays the corresponding irradiance levels inside the vehicles. A statistical analysis of the UV irradiance data is presented in Table 1.

**Table 1:** The UV intensity inside and outside the vehicle during sunny days of June 2021.

**Measurement site**	**UV intensity (mW/cm** ^**2**^ **)**			
	**Maximum**	**Minimum**	**Mean**	**STD**
Outside vehicle	5.90	2.39	3.92	1.37
Inside vehicle (front window)	0.80	0.21	0.32	0.15
Inside vehicle (side window)	1.70	0.26	0.82	0.34

### 3.3 IR irradiance levels

**Figure 7:**
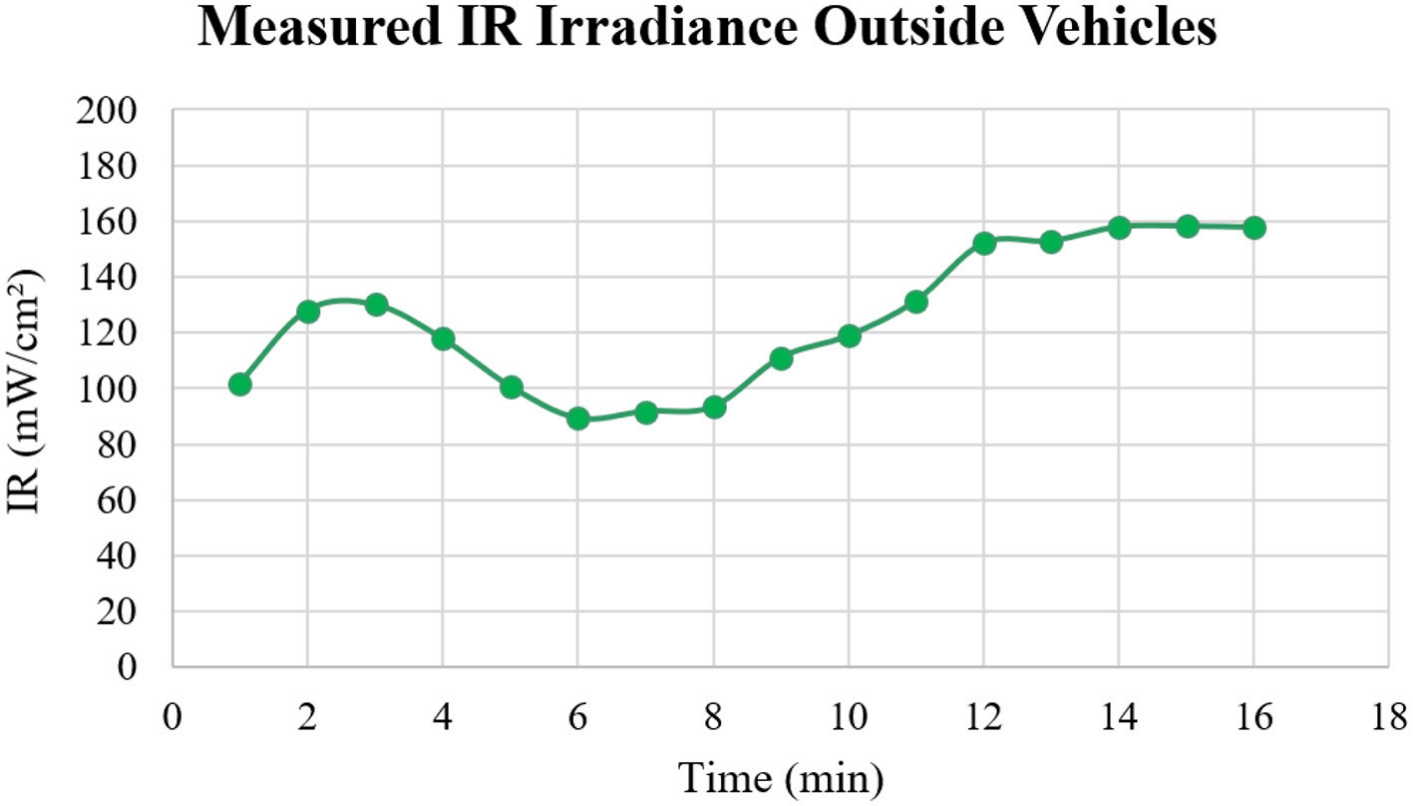
IR measurements outside the vehicles for 16 min.

**Figure 8:**
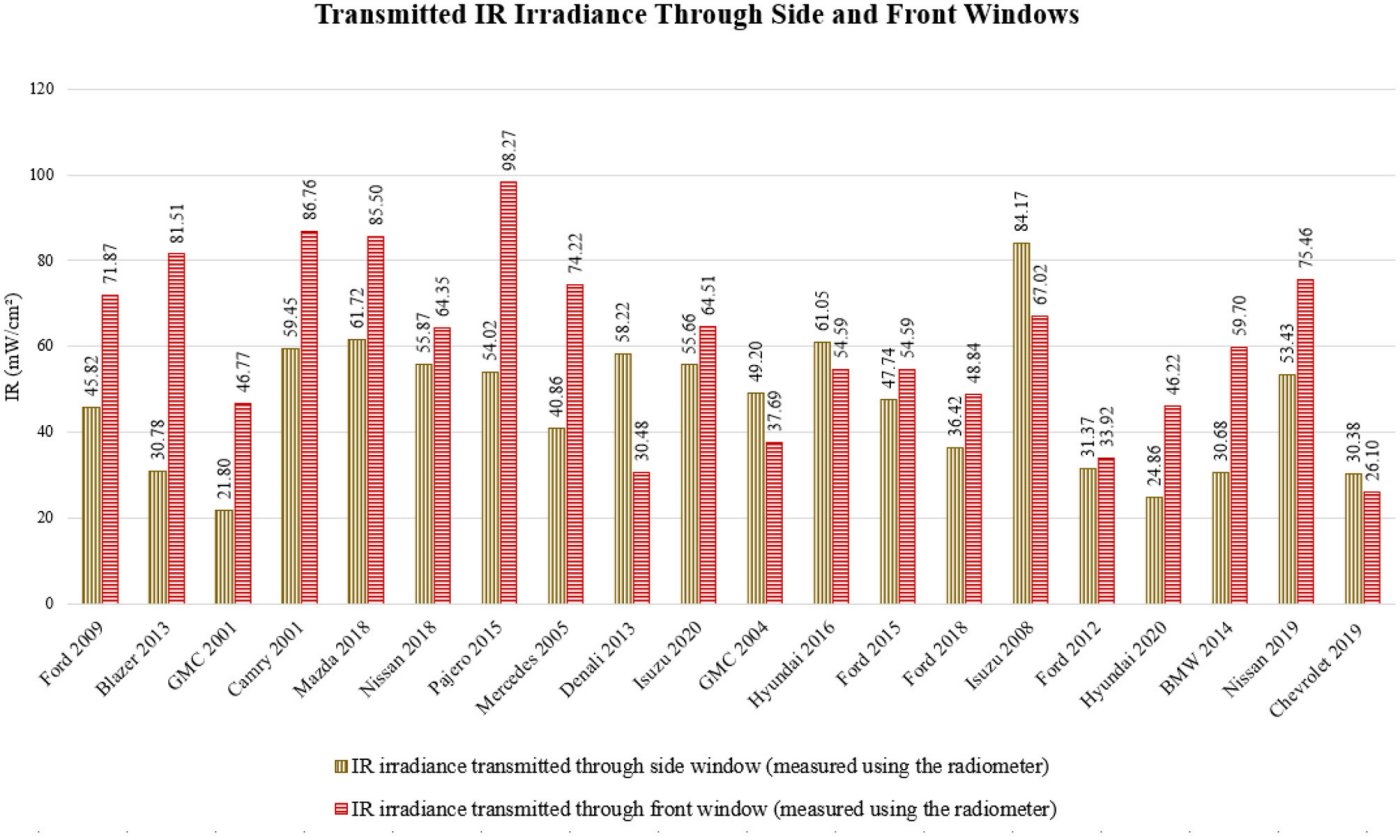
Transmitted IR irradiance through the side (represented in orange color) and front (represented in yellow color) windows in 20 vehicle models; the measurements were taken using the radiometer.

IR irradiance levels were measured to assess thermal exposure both inside and outside the vehicles. Significant differences in IR irradiance were observed across the vehicles, similar to the variations noted in the UV irradiance measurements. Figure 7 illustrates the IR irradiance levels measured outside the vehicles, while Figure 8 presents the corresponding levels inside. A detailed statistical analysis of the IR irradiance data is provided in Table 2.

**Table 2:** The IR intensity inside and outside the vehicle during sunny days of June 2021.

**Measurement site**	**IR intensity (mW/cm** ^**2**^ **)**		
	**Maximum**	**Minimum**	**Mean**	**STD**
Outside vehicle	158.33	89.24	124.58	25.42
Inside vehicle (front window)	98.27	26.10	60.42	20.14
Inside vehicle (side window)	84.17	21.80	46.68	15.65

## 4 Discussion

Moderate sun exposure is essential for the synthesis of vitamin D, which is crucial for bone health and immune function. However, excessive exposure to solar radiation, particularly UV radiation, presents significant health risks, including DNA damage in skin cells, sunburn, premature aging, and an increased risk of skin cancers such as melanoma. The challenge lies in balancing the beneficial effects of sunlight with the need to mitigate its harmful consequences. To address this, well-established sun safety practices, such as applying sunscreen, wearing protective clothing, and avoiding direct sunlight during peak hours, are widely recognized as effective strategies for minimizing risks. By adhering to these preventive measures, individuals can obtain the health benefits of sun exposure while reducing the potential for skin damage (16). As complete avoidance of solar radiation, especially during peak sunlight hours, is often impractical for outdoor workers and drivers, mitigating such exposure is essential. This investigation assesses the potential risks associated with UV and IR radiation exposure inside vehicles, emphasizing the critical need for enhanced protective measures against solar radiation penetration through vehicle windows. This study advocates for a paradigm shift in vehicle safety design to protect vehicle occupants from the long-term health risks associated with prolonged exposure to solar radiation, particularly with regard to skin damage and an increased risk of skin cancer.

The survey results, depicted in Figure 4, reveal a significant yet often underestimated awareness regarding UV and IR radiation exposure inside vehicles. While 82% of participants reported awareness of the ability of UV and IR radiation to penetrate vehicle windows, and 88% recognized the risks of solar exposure, there was a noticeable gap in the implementation of safety practices. For example, although 82% of participants make efforts to avoid direct sunlight, only 44% apply sunscreen despite being aware of the risks associated with UV and IR exposure. This discrepancy suggests that while there is some recognition of the risks, preventive measures, such as sunscreen use, are not consistently considered, potentially leading to prolonged exposure that could increase the risk of skin damage.

When UV and IR radiation levels were measured outside the vehicles, the results indicated a steady increase in UV radiation levels, ranging from 2.39 to 5.90 mW/cm^2^, as shown in Figure 5. This upward trend emphasizes the cumulative nature of solar radiation exposure, suggesting that prolonged exposure can lead to progressively higher UV radiation levels over time. Prolonged and uncontrolled exposure to UV radiation significantly elevates the risk of skin damage and long-term health consequences, including skin cancer. Conversely, the IR radiation measurements, shown in Figure 7, exhibited considerable variability, ranging from 89.24 to 158.33 mW/cm^2^. These fluctuations are likely due to environmental factors, such as the movement of clouds, which intermittently block sunlight and absorb some of the IR radiation. The variability underscores the dynamic nature of IR exposure, further complicating efforts to predict and manage exposure levels. A study by Moehrle et al. (21), reported that UV exposure accounted for ~3 to 4% of ambient radiation when car windows were closed. In contrast, our study found UV exposure to be significantly higher, ranging from 13.56 to 28.81%. This difference is likely due to the hot climate of Dammam, which may result in greater solar radiation penetration through the car windows. Another study by Parisi et al. (18) in Australia found that annual solar UVA exposure ranged from 5 to 17% of the ambient UVA on a horizontal plane. However, this range decreased to 1–8% after tinted films were applied to the vehicle windows.

The analysis of UV transmission through vehicle windows revealed distinct patterns between different vehicle models, as illustrated in Figure 6. For instance, the 2001 Toyota Camry exhibited the highest UV radiation transmission through its front window at 0.80 mW/cm^2^, while several other vehicles, including the 2009 Ford, 2013 Blazer, 2018 Mazda, 2015 Pajero, 2005 Mercedes, 2013 Denali, 2012 Ford, and 2019 Chevrolet, showed the lowest transmission at 0.21 mW/cm^2^. These variations in UV transmission are likely attributed to the specific manufacturing processes and design characteristics of the windows. Specifically, side windows, which are typically made of tempered glass, allow more UV radiation to pass through compared to the laminated glass used in front windows, which is designed to block a significant portion of UV radiation. Vehicles like the 2001 Toyota Camry and 2008 Isuzu displayed the highest UV transmission through their side windows, with measurements of 1.55 and 1.70 mW/cm^2^, respectively. This increased transmission is likely due to the less effective PVB layer used in side windows, which permits greater UV radiation penetration. These findings are consistent with prior studies that have shown UV radiation can penetrate vehicle windows (6, 18, 21).

The IR radiation measurements, presented in Figure 8, revealed that the 2008 Isuzu recorded the highest IR radiation transmission through its front window at 98.27 mW/cm^2^, while the 2015 Mitsubishi Pajero exhibited the highest IR transmission through its side windows at 84.17 mW/cm^2^. These findings suggest that IR radiation can penetrate both side and front windows, especially when IR coatings are absent. This observation is consistent with theoretical studies indicating that IR radiation may pass through vehicle windows, particularly in the absence of IR coatings (23). The lack of IR protection in vehicle design is likely due to cost considerations, as UV protection has historically received more attention due to its stronger association with skin cancer risks.

Statistical analysis of UV and IR radiation intensities, summarized in Tables 1, 2, revealed that the average UV radiation exposure outside vehicles was approximately seven times higher than inside, underscoring the influence of vehicle window design on UV transmission levels. Conversely, IR radiation outside the vehicles was approximately two times higher than inside. Although UV radiation has been extensively studied in the context of vehicle window design, IR radiation transmission through vehicle windows has received far less attention, highlighting a critical gap in vehicle design. The results of this study emphasize the need for more comprehensive window designs that integrate both UV and IR protection technologies to protest vehicle occupants from harmful solar radiation.

While the effects of UV radiation on skin are well-documented, the impact of both UV and IR radiation on skin color should also be considered. Individuals with lighter skin tones are more susceptible to UV-induced skin damage, such as sunburn and increased risk of melanoma, due to lower levels of melanin, which provides some natural protection. In contrast, darker-skinned individuals have more melanin, which offers greater protection against UV radiation but does not provide immunity. In terms of IR radiation, all skin colors may experience thermal effects. Lighter skin can feel discomfort more acutely due to its lower melanin content and lower tolerance for heat, while darker skin may experience less visible thermal irritation but can still suffer from underlying tissue damage, particularly with extended exposure to high levels of IR radiation (27–31). This highlights the need for protective strategies for individuals of all skin types, as both UV and IR radiation can cause long-term skin damage and health risks.

The findings of this study emphasize the significant risks associated with both UV and IR radiation penetration through vehicle windows. Despite some level of UV protection provided by laminated glass, our results demonstrate that considerable levels of UV radiation continue to penetrate these materials, especially through side windows. This is particularly concerning for individuals who spend extended periods inside vehicles. Moreover, IR radiation exposure, which has been less studied, represents a significant concern, particularly in vehicles without effective IR coatings. Therfore, addressing these concerns is crucial for improving the safety and wellbeing of vehicle occupants and raising awareness about the often-overlooked risks of solar radiation exposure inside vehicles. This study recommends further research and development in vehicle window design to enhance protection for both drivers and passengers against long-term skin damage and associated health risks.

## 5 Conclusion

The benefits of solar radiation are widely acknowledged; however, it also emits harmful UV and IR radiation that reaches the Earth's surface. Prolonged exposure to these types of radiation can result in adverse skin effects, including sunburn, premature photoaging, and an increased risk of skin cancer. A survey involving 1,293 participants was conducted to evaluate public awareness regarding solar radiation and the protective behaviors individuals engage in to mitigate its effects. While a majority of respondents recognized the risks associated with solar exposure, only a minority consistently employed sunscreen or implemented other preventive measures to reduce exposure.

This study quantified the extent to which UV and IR radiation penetrate vehicle windows and evaluated the level of protection offered by various materials and manufacturers. The findings highlighted the necessity of developing effective solutions to mitigate exposure to these harmful rays within the vehicular environment. A data logger radiometer was used to measure UV and IR radiation levels inside 20 different vehicles. The results demonstrated that UV and IR radiation levels inside vehicles were sufficiently high to cause potential skin damage, particularly with extended Exposure.

Outdoor UV radiation levels ranged from 2.39 to 5.90 mW/cm^2^, with a consistent increase over time, while IR radiation levels fluctuated between 89.24 and 158.33 mW/cm^2^. UV transmission through vehicle windows demonstrated significant variability across different models. For example, the 2001 Toyota Camry exhibited the highest UV transmission through its front window, measuring 0.80 mW/cm^2^, while the 2008 Isuzu allowed the highest UV transmission through its side windows, with a reading of 1.70 mW/cm^2^. Additionally, the 2008 Isuzu showed the highest IR transmission, with 98.27 mW/cm^2^ recorded through its front window. In contrast, the 2015 Mitsubishi Pajero displayed the highest IR transmission through its side windows, at 84.17 mW/cm^2^.

These results reveal a significant gap in current automotive window design, emphasizing the urgent need for solutions that provide protection from both UV and IR radiation. The study recommends continued research and development in vehicle safety technologies to mitigate the long-term health risks, including skin damage, associated with exposure to solar radiation.

## Data Availability

The datasets that were utilized and examined in this study can be obtained from the corresponding author upon reasonable request.
